# Role of nicergoline in corneal wound healing in diabetic rats

**DOI:** 10.1186/s12886-021-01835-4

**Published:** 2021-02-09

**Authors:** Amanda Lemos Barros Martins Portela, Rafael Neves Moreno, Maria Helena Madruga Lima Ribeiro, Fernanda Miguel de Andrade, Yale Viana Alves, Mônica Alves, Rodrigo Pessoa Cavalcanti Lira

**Affiliations:** 1 Federal University of Pernambuco (UFPE), Recife, Pernambuco Brazil; 2 Keizo-Asami Immunopathology Laboratory (LIKA), Recife, Pernambuco Brazil; 3 Federal University of Campina Grande (UFCG), Campina Grande, Paraíba Brazil; 4 University of Campinas (UNICAMP), Campinas, São Paulo, Brazil

**Keywords:** Corneal epithelium, Diabetes mellitus, Diabetic neuropathy, Nicergoline, Wound healing, Corneal ulcer

## Abstract

**Background:**

To investigate the effect of nicergoline on the rate of complete corneal ulcer reepithelialization (CCUR) in diabetic rats with diabetic keratopathy.

**Methods:**

Forty-eight streptozotocin-induced diabetic rats were randomly divided into two groups. The experimental group (*n* = 24) received nicergoline (10 mg.kg^− 1^.day^− 1^), while the control group (n = 24) received a placebo. A corneal epithelial defect was induced using a corneal diamond burr, and defect area was compared at time points of 0, 12, 24, 48 and 72 h after the injury using image analysis software. The probability of CCUR within 72 h was assessed using the Kaplan–Meier survival analysis log-rank test.

**Results:**

When compared, 4 of the 24 rats (17%) in the placebo group and 12 of the 24 rats (50%) in the nicergoline group were found to have CCUR within 72 h (log-rank = 0.027). Cox regression analysis found no effect of the covariates blood glucose (*P* = 0.601) or weight (*P* = 0.322) on the corneal reepithelialization (survival) curve.

**Conclusions:**

Nicergoline increased wound healing rates relative to placebo and may therefore be investigated as a treatment option in diabetic keratopathy.

## Background

Diabetes affects 451 million people worldwide, approximately 70% of whom suffer from corneal complications known collectively as diabetic keratopathy. The most frequent clinical signs of diabetic keratopathy include punctate keratitis, recurrent epithelial erosions, and ulcers that are refractory to conventional treatments and which often fail to heal completely [1].

Previous controlled studies with corneal epithelial cell cultures in medium with high glucose levels have revealed that hyperglycemia over a period of 48 h is able to delay corneal reepithelialization to levels of only 29% of normal and to greatly increase the production of reactive oxygen species [2]. The pathogenesis of diabetic keratopathy is multifactorial; prior studies have pointed to the impairment of the phosphatidylinositol 3-kinase (PI3K/AKT) transduction pathway, which responds to cell proliferation and migration in various systems and which also affects corneal healing [2]. In addition, oxidative stress and inflammatory cytokine production generated by hyperglycemia greatly contribute to the induction of cellular injury [2, 3].

In a previous study, rats with streptozotocin-induced diabetes exhibited a significant reduction in subepithelial basal nerve plexus density, as well as delayed closure of corneal epithelial ulcers 8 weeks after diabetes induction relative to controls [4].

Certain growth factors, cytokines, and neurotrophins are fundamental to corneal wound healing and act as regulators of cellular behavior and corneal epithelial regeneration. These include epidermal growth factor (EGF), fibroblast growth factor (FGF), substance P (SP), nerve growth factor (NGF), insulin-like growth factor-1 (IGF-1), and acetylcholine [5, 6]. New drugs targeting such factors have been investigated to treat diabetic keratopathy, since diabetes is associated with a significant decrease in the levels of these molecules [7–12].

Nicergoline (10a-methoxy-1,6-dimethylergoline-8β-methanol-5- bromonicotinate, Sermion, Biogenesis AntiAging, Fish Hoek, South Africa) is an ergoline derivative indicated for cerebrovascular dementias with a broad mechanism of action. It acts as an α-1 adrenergic antagonist, acetylcholinesterase inhibitor, dopaminergic agonist and PI3K/AKT pathway activator; it also increases SP and NGF levels and exhibits antioxidant properties. Such properties have inspired research into its use in corneal wound healing [13–18].

In a previous study, nicergoline contributed to the corneal reepithelialization of 110 eyes from 100 rats, and corneal NGF protein was higher in the nicergoline-treated group than in the control group [16]. In another experimental study that assessed 10 eyes from 5 healthy dogs without a control group, the effect of nicergoline on the ocular surface was assessed using corneal esthesiometry, Schirmer’s test 1, and tear film break-up time, but the drug did not significantly alter canine ocular surface parameters [19].

In a prospective, noncomparative interventional study that included 27 eyes from 24 patients with neurotrophic keratopathy of multiple causes who had been unresponsive to conventional therapy, treatment with nicergoline contributed to the healing of neurotrophic corneal ulcers in 83% of eyes and improved Cochet–Bonnet corneal sensitivity and best-corrected visual acuity. In addition, tear NGF levels were significantly higher after nicergoline treatment [20].

Given the evidence that nicergoline may improve corneal healing processes in healthy animals, this study sought to evaluate the healing potential of nicergoline for the corneas of diabetic rats, and to assess its capacity to support the complete reepithelialization of corneal ulcers secondary to diabetic keratopathy.

## Methods

### Ethical statements

This study was approved by the Research Ethics Committee on Animal Research (CEUA) of the Federal University of Pernambuco (UFPE) and it was carried out in compliance with the ARRIVE guidelines. All procedures considering animals in this study adhered to the ARVO resolution for the care and use of animals in visual research.

### Experimental model

The animals were acquired from the vivarium of the Department of Nutrition of the Federal University of Pernambuco and were housed in the vivarium of the Keizo-Asami Immunopathology Laboratory (LIKA).

An experimental, controlled, and double-blind study design was employed with 48 male Wistar rats *(Rattus norvegicus*) between 8 and 10 weeks of age and weighing between 200 and 300 g. They had no genetic modifications and had received all relevant vaccinations.

All animals had free access to appropriate feed and water and were housed in cages (4 rats per cage) with sawdust bedding under controlled temperature conditions (24 °C ± 1 °C) and with a 12 h light–dark cycle (light switched on at 8:00 AM and switched off at 8:00 PM).

### Induction of diabetes mellitus

The animals were housed in cages for 1 week before the start of the diabetes induction procedure. A single dose of 60 mg.kg^− 1^ of streptozotocin (Sigma-Aldrich, St. Louis, MO) in a 0.5 M sodium citrate buffer solution at a pH of 4.5 (Alfa Aesar Citrate, Fischer Scientific, Hampton, New Hampshire, USA) was administered through intraperitoneal injection [21].

In this experiment, 1 ml syringes (26G) were used for the intraperitoneal injection of the streptozotocin buffer solution 12 h after food withdrawal (the animals continued to receive free access to water during this fasting period). As previous protocols have established, the induction of diabetes by streptozotocin triggers minimal pain and therefore does not require anesthesia [22].

Fifteen days after induction, each animal’s weight and blood glucose levels were monitored periodically. Diabetes was confirmed by measuring blood glucose from a blood sample from the caudal vein with glucometer (Free Style Lite, Abbott, Chicago, Illinois, USA) 8 h after fasting. Diabetes was diagnosed if blood glucose was greater than or equal to 200 mg.dl^− 1^. Forty-eight diabetic animals were included in the study and randomly divided into the experimental nicergoline group (*n* = 24) and the placebo control group (n = 24).

### Nicergoline treatment

The 48 diabetic rats were treated by a blinded researcher 6 weeks after diabetes induction. The experimental group received nicergoline at a dosage of 10 mg.kg^− 1^.day^− 1^ that was diluted in each rat’s drinking water. Meanwhile, the placebo group received drinking water without nicergoline. The animals’ drinking water was routinely acidified using HCl to avoid the growth of pathogenic microorganisms; this acidification process is a sanitation measure typically used in vivariums and one which is safe for laboratory animals [16–18].

Oral administration was performed in both groups using gavage and manual containment 2 weeks prior to the corneal injury procedure, a protocol which was based on prior studies with a similar scope [16].

Radioisotope-marked nicergoline reaches peak serum radioactivity 3 h after oral administration. Its bioavailability is 5% of the total dose administered due to the first-pass effect, and it is predominantly eliminated in the urine. On average, 82% of marked nicergoline is eliminated in the urine and 10% in the feces as of 120 h after administration [19, 23].

Nicergoline was not administered in the 72-h postoperative period; during this time, the animals were under periodic anesthesia/sedation for clinical evaluation and data collection.

### Corneal wound procedure

Immediately after treatment (nicergoline or placebo), the animals were anesthetized through the intramuscularly administration of 10% ketamine hydrochloride (Cetamin, Syntec, São Paulo, Brazil) at a dose of 50 mg.kg^− 1^ and 2% Xylazine Hydrochloride (Xilazin, Syntec, São Paulo, Brazil) at a dose of 10 mg.kg^− 1^; they also received a topical application of 0.4% oxybuprocaine hydrochloride eye drops (Oxinest, Cristalia Prod. Quim., Farm. Ltda, São Paulo, Brazil) in the left eye (OS). After anesthesia, superficial keratectomy surgery was performed on the left eyes by a blinded researcher.

The size of the corneal epithelial injury to be made was pre-established by using a corneal trephine 3.0 mm in diameter (Odous Instrumentos Ltda, Contagem, MG, Brazil). The area bound by the trephine was then de-epithelialized with a corneal diamond burr (AlgerBrush II, Alger company, Lago Vista, TX), thus generating a central corneal defect, as described previously [4, 16, 24].

### Evaluation of corneal Reepithelialization

To monitor corneal reepithelialization, the animals were anesthetized following the same procedure described previously and were evaluated at predetermined time points of 0, 12, 24, 48, and 72 h by a blinded researcher. The epithelial defect was evaluated by staining with 1% sodium fluorescein under cobalt blue filter and photographed using a Canon EOS Rebel T5i/EOS 700 D digital camera attached to the Opto SM Plus IBZ surgical microscope using an adapter. The area of the epithelial defect was measured by a different blinded researcher through photo analysis using Adobe Photoshop [4, 16, 25].

Seventy-two hours after the procedure, the animals were euthanized using 3% sodium pentobarbital (Hipnol, Syntec, São Paulo, Brazil) at an intramuscular dose of 150 mg.kg^− 1^ and monitored until heartbeats stopped.

### Statistical analysis

Descriptive statistic data were calculated. The Kolmogorov–Smirnov normality test was used for continuous data. Means and standard deviations (SDs) were used for normally distributed data, and medians and interquartile ranges (IQR) were used for non-normally distributed data. Between-group differences in continuous variables were compared using the independent samples t-test for normally distributed data or using the Mann–Whitney U-test for non-normally distributed data.

The probability of reach complete corneal ulcer reepithelialization (CCUR) within 72 h was assessed using the Kaplan–Meier survival analysis log-rank test. Multivariate survival analysis was performed using the Cox regression model. The analyses were conducted using SPSS, version 21 (IBM Corporation, Armonk, NY, USA). *P*-values were two-tailed, and statistical significance was set at 0.05.

## Results

Demographic data are displayed in Table 1 and demonstrate homogeneity of the two groups in terms of weight and blood glucose.

**Table 1:** Demographic data of the diabetic rats in the placebo and nicergoline groups

Clinical Value	Placebo (***n*** = 24)	Nicergoline (***n*** = 24)	***p***-value
Blood glucose,^a^ mean (SD^c^), median (IQR^d^), mg/dL	351 (81), 308 (156)	314 (66), 309 (95)	0.085 ^b^
Weight,^a^ mean (SD), median (IQR), g	230 (39), 230 (68)	247 (34), 250 (53)	0.103 ^b^

^a^normally distributed data; ^b^independent samples t-test, ^c^ standard deviation, ^d^interquartile range

The analyses revealed that 4 of the 24 rats (17%) in the placebo group and 12 of the 24 rats (50%) in the nicergoline group achieved complete reepithelialization of the cornea within 72 h (log-rank = 0.027). Cox regression analysis showed no influence of covariates on the survival curve of the corneal reepithelialization variable. The covariates assessed were glycemia (*P* = 0.601), weight (*P* = 0.322), and duration of diabetes (*P* = 0.208).

Table 2 details corneal ulcer reepithelialization progress. The area of the corneal ulcer at the 0-h time point (immediately after surgery) was found to be consistent between the two groups: 6.42 ± 1.17 mm^2^ in the placebo group and 6.07 ± 1.31 mm^2^ in the nicergoline group (*P* = 0.330).

**Table 2:** Corneal reepithelialization in diabetic rats that underwent mechanical de-epithelialization after treatment with nicergoline or placebo

Measurement Time	Epithelial Defect Area: mean (SD^**c**^), median (IQR)^**d**^ in mm^**2**^		***p***-value
	Placebo (***n*** = 24)	Nicergoline (***n*** = 24)	
0 h ^a^	6.42 (1.17), 6.20 (1.28)	6.07 (1.31), 6.07 (1.27)	0.330 ^e^
12 h ^a^	5.69 (0.99), 5.40 (0.78)	4.71 (1.53), 4.68 (2.51)	0.018 ^e^
24 h ^a^	4.52 (1.56), 4.45 (2.36)	3.15 (1.65), 3.30 (3.08)	0.012 ^e^
48 h ^b^	3.57 (3.21), 1.67 (4.52)	2.07 (1.41), 0.50 (2.00)	0.095 ^f^
72 h ^b^	3.51 (3.48), 0.97 (4.38)	1.24 (0.87), 0.00 (0.72)	0.017 ^f^

^a^normally distributed data, ^b^non-normally distributed data, ^c^standard deviation, ^d^interquartile range, ^e^independent samples t-test, ^f^Mann–Whitney U-test

Table 2 also shows that the area of the corneal ulcer was found to be significantly smaller in the nicergoline group at various time points. At the 12-h time point, the area of the corneal ulcer in the nicergoline group (4.71 ± 1.53 mm^2^) was smaller than that of the placebo group (5.69 ± 0.99 mm^2^; *P* = 0.018).

The ulcers in the experimental group were also smaller at the 24-h time point: 3.15 ± 1.65 mm^2^ in the nicergoline group compared to 4.52 ± 1.56 mm^2^ in the placebo group (*P* = 0.012).

The findings were consistent at the 72-h time point as well, at which point the area of the corneal ulcer in the nicergoline group (1.24 ± 0.87 mm^2^) was considerably lower than that of the placebo group (3.51 ± 3.48 mm^2^; *P* = 0.017).

**Fig. 1:**
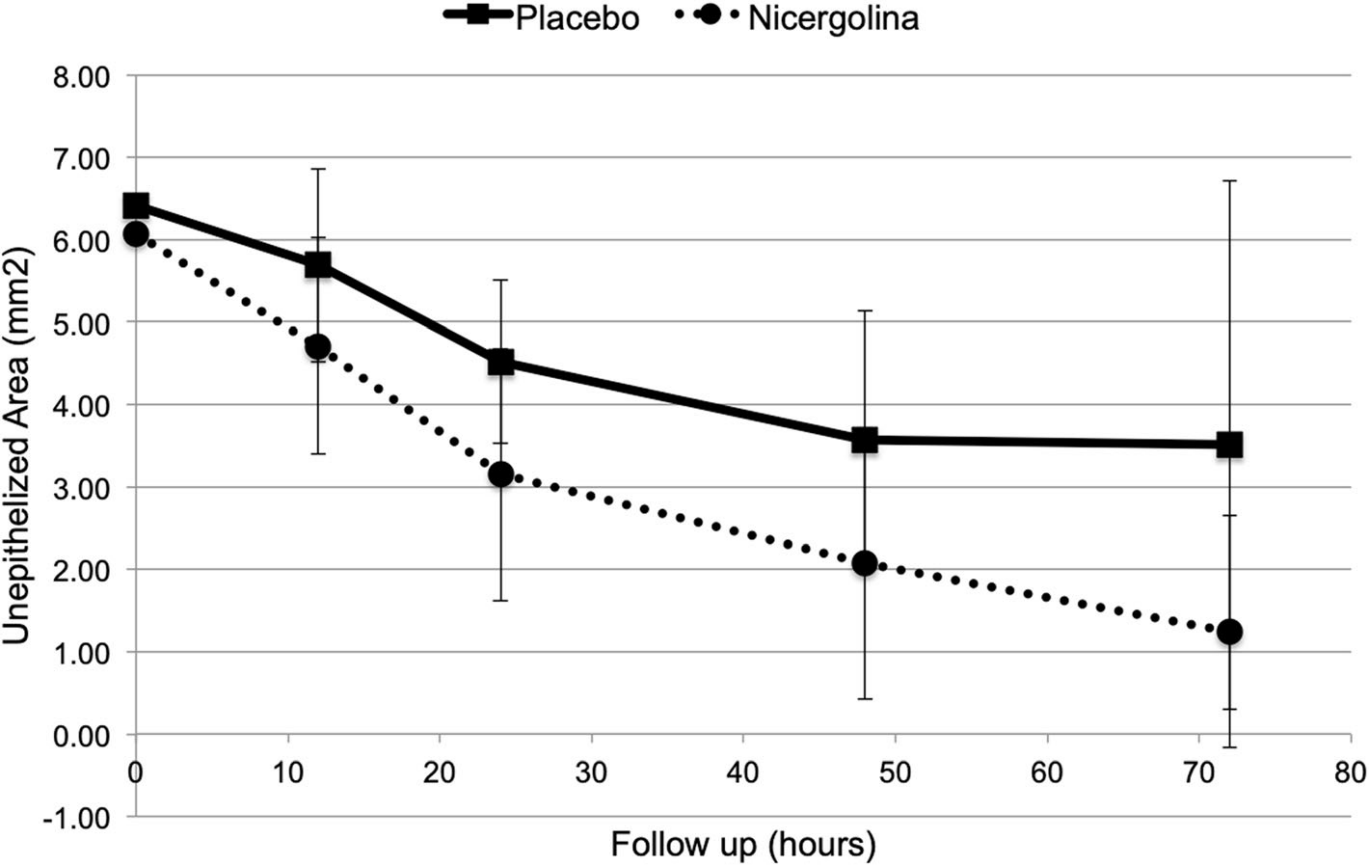
Corneal reepithelialization progress in diabetic rats along the time points considered in eyes treated with nicergoline (*n* = 24) or placebo (*n* = 24)

**Fig. 2:**
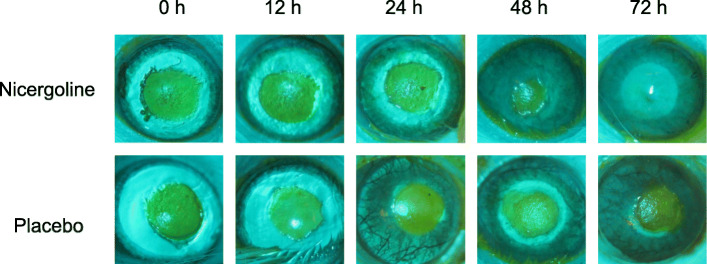
Images of fluorescein corneal staining showing the area of epithelial defect in green along the time points considered in eyes treated with nicergoline or placebo. The images are of an eye of a single animal from each study group

Figure 1 illustrates the corneal reepithelialization process in the two groups. Figure 2 shows an example of 1% fluorescein staining of the corneal epithelial defect in the nicergoline and placebo groups immediately after surgery and at the 12-, 24-, 48-, and 72-h time points thereafter.

The median and interquartile range (IQR) of the reepithelialization rate in the first 72 h was 0.04 (0.06) mm^2^/h in the placebo group and 0.08 (0.03) mm^2^/h in the nicergoline group (*P* = 0.055).

## Discussion

The present study relied on an experimental model that reproduced insulin-dependent diabetes type 1. The animals presented epithelial defects that failed to heal completely, thus resulting in the corneal ulcers commonly seen in cases of diabetic keratopathy.

Reepithelialization occurred twice as quickly in the experimental group as in the control group. Similar studies have found a 1.55-fold increase in mean reepithelialization speed using a fibronectin-derived peptide eye drop treatment and a 1.47-fold increase using SP with insulin-like growth factor-1 eye drop treatment [24].

The two groups were also compared in terms of their ability to reach CCUR. Nicergoline was associated with significantly more corneal ulcer healing: after 72 h, 50% of the nicergoline group presented CCUR, while only 17% the of placebo group exhibited the same outcome (log-rank = 0.027). Previous studies have also reported a delay in the reepithelialization of corneal defects in diabetic rats and a persistence of unhealed epithelial defects after 72 h, and, in other studies, after more than 120 h [10, 25].

The nicergoline group exhibited a statistically significant decrease in the area of the corneal ulcer at the 12-h, 24-h, and 72-h time points. This finding suggests that nicergoline promotes corneal epithelial healing in diabetic rats and may be considered a promising new treatment option worthy of further investigation.

To date, this is the first experimental, controlled, and double-blind study to investigate the potential of nicergoline to improve the rate of corneal reepithelization and to treat corneal ulcers in a clinical context of diabetes, since the other studies in the literature evaluated this drug only in healthy animals [13, 14, 20].

The current study exhibits some limitations. It is important to mention the lack of proof of diabetic neuropathy by an objective test, such as the corneal sensitivity test with the Cochet–Bonnet esthesiometer. We opted to forgo this procedure due to its low sensitivity and reproducibility [26, 27].

A second limitation was the type of intervention used (nicergoline vs. placebo) prior to corneal surgical injury. Nicergoline is a macromolecule that is currently only administered via the oral, intravenous, and intramuscular routes. It exhibits poor solubility in water (0.002 mg.mL^− 1^ at 25^ο^C) in its commercial crystalline form; its hydrophobic and lipophilic properties therefore hinder its penetration into the hydrophilic corneal stroma, and its molecular size limits its ability to pass through the apical tight junctions of the epithelium. To resolve this problem, hydrolysis reactions may be used to modify the polarity of the drug; even so, the size of the molecule remains a limiting factor. Another solution is to change the size of nicergoline molecules through the use of nanoparticle assembly processes, which can reduce molecular size and allow for nanocrystals to be produced in an aqueous dispersion medium and stabilized using a surfactant or polymer [28].

Recent studies have reported on the use of nicergoline in the form of nanoparticles to be administered solely via the oral route; this process has been found to increase its solubility and, as a result, its bioavailability, which is low (5%) in the drug’s commercial crystalline form. This type of pharmacological nanotechnology has been applied to certain anti-glaucoma drugs produced as eye drops, but it has not been applied to nicergoline as an eye drop. Thus, the use of nicergoline nanocrystals is currently limited to the oral route; a nicergoline eye drops were not available yet to be employed in studies. The findings in this study may inspire further research in the field of pharmacological technology in order to develop nicergoline-based nano eye drops [29, 30].

The animals’ drinking water was suspended with nicergoline. To compensate for the drug’s low solubility in water and consequently limited bioavailability, it was administered at a loading dose 2 weeks prior to the corneal injury procedure in order to prolong the animals’ time of exposure to the drug. The nicergoline dosing regimen used in this study was similar to the methodology used in previous studies and was therefore considered appropriate [16, 29, 30].

Another limitation was that post-treatment serum SP and NGF levels in the corneal tissue of the animals were not measured, but as described previously, an increase in SP and NGF in corneal tissue after nicergoline treatment has been previously observed in healthy animals [16, 17].

## Conclusions

In conclusion, nicergoline was found to be associated with corneal wound healing in diabetic rats and likely contributed to the outcomes found herein. The rate of complete corneal ulcer reepithelialization after 72 h was higher among diabetic rats receiving nicergoline than in the placebo group. This evidence is promising, and more studies are needed to evaluate nicergoline as a potential treatment of corneal ulcers secondary to diabetic keratopathy.

## Data Availability

The datasets used and/or analysed during the current study are available from the corresponding author on reasonable request.

## Abbreviations

CCUR: Complete corneal ulcer reepithelialization
PI3K/AKT: Phosphatidylinositol 3-kinase AKT pathway
EGF: Epidermal growth factor
FGF: Fibroblast growth factor
SP: Substance P
NGF: Nerve growth factor
IGF-1: Insulin-like growth factor-1
CEUA: Research Ethics Committee on Animal Research
UFPE: Federal University of Pernambuco
CONCEA: National Council for the Control of Animal Experimentation
LIKA: Keizo-Asami Immunopathology Laboratory
OS: Left eye
SDs: Standard deviations
IQR: Interquartile range
